# Effects of combined application of slow-release nitrogen fertilizer and urea on nitrogen uptake, utilization and yield of maize under two tillage methods

**DOI:** 10.1038/s41598-025-87480-z

**Published:** 2025-02-11

**Authors:** Mingyuan Fan, Pei Chen, Chang Zhang, Min Liang, Guangming Xie, Li Zhao, Chuangyun Wang

**Affiliations:** 1 College of Agronomy, Shanxi Agricultural University, 030031 Taiyuan, Shanxi China; 2 Shanxi Agricultural University, 030031 Taiyuan, Shanxi China

**Keywords:** Farming practices, Fertilizer application, Dry matter accumulation, Nitrogen absorption, utilization and operation, Yield, Physiology, Plant sciences

## Abstract

Corn is one of the important food crops in the world, in order to pursue high yield and high efficiency, the input of chemical fertilizer has been continuously increased, which has led to the decline of fertilizer utilization rate, environmental pollution and other problems, and the slow-release fertilizer has attracted much attention because of its nutrient characteristics, but the high price limits its wide application;On the other hand, long-term rotary tillage will lead to the shallowness of the soil tillage layer and the increase of the bulk density of the deep soil, and subsoiling can effectively break the bottom layer of the soil plough and reduce the bulk density of the soil. Therefore, in this study, Qiangsheng 388 was used as the experimental material, and under rotary tillage (R) and rotary tillage + subsoiling (R+S), CK (no fertilization), U (100% nitrogen fertilizer), S (100% slow-release fertilizer) and different UNS ratios (nitrogen fertilizer and slow-release fertilizer) were used for two years in field experiments to explore the effects of slow-release fertilizer and urea on soil water use efficiency, dry matter accumulation, nitrogen uptake and utilization, operation and yield under the two tillage methods. The results showed that compared with CK, U and S, UNS significantly improved water use efficiency, dry matter accumulation, nitrogen uptake, operation and yield. Among them, UNS2 (the ratio of S and U was 7:3) was the best. In 2022, R UNS2 had the best water use efficiency, dry matter accumulation, nitrogen uptake, operation and yield, which were 5.05%, 2.0%, 3.88%, 6.18% and 4.06% higher than those of R + S UNS2, respectively. In 2023, R + S UNS2 had the best treatment, which was 1.98%, 2.52%, 1.85%, 2.60% and 2.20% higher than that of R UNS2, respectively. R + S tillage can effectively improve maize yield, nitrogen uptake and utilization, water use and dry matter accumulation; Nitrogen application treatment UNS2 treatment was the best. In summary, R + S UNS2 is the best coordination strategy.

## Introduction

Nitrogen is an important nutrient for the growth and development of crops. Therefore, the application of nitrogen fertilizer has become one of the main measures to increase agricultural yield^1,2^. In recent years, in order to further improve crop yields, the amount of nitrogen fertilizer application in China has increased year by year^3^. However, the linear increase in N input did not lead to a significant increase in crop yields, but instead led to a sharp decrease in N use efficiency (NUE)^4,5^ and environmental damage. In order to increase crop yields and reduce costs without further increasing the use of chemical fertilizers, a series of optimized nitrogen fertilizer management measures were proposed. Compared with conventional fertilizers, slow-release nitrogen fertilizers can improve crop yield and nitrogen use efficiency by slowly releasing nutrients according to crop needs, improving fertilizer timeliness and reducing the number of fertilization applications^6–9^. In recent years, slow-release nitrogen fertilizers have been studied and applied in improving crop yield and nitrogen use efficiency^10,11^. Zhou et al.^12^ concluded that compared with conventional nitrogen fertilizer, slow/controlled-release fertilizer can significantly improve maize yield and nitrogen use efficiency. Garcia et al.^13^ and Gautam et al.^14^ showed that compared with ordinary chemical fertilizers, slow-release nitrogen fertilizers could effectively control the nitrogen release rate, effectively provide nitrogen sources in the later stage of crop growth, and had significant effects on increasing crop dry weight and nitrogen accumulation. Tian et al.^15^ showed that slow-release nitrogen fertilizer could reduce nitrogen application rate without reducing crop yield and improve nitrogen use efficiency. He et al.^16^ showed that the application of slow-release nitrogen fertilizer not only promoted the nitrogen supply in the late growth period of crops, but also effectively increased nitrogen conversion and accumulation, coordinated the nutrient absorption and distribution of various organs of crops, and greatly improved crop yield and nitrogen use efficiency. However, the cost increases due to the high price of slow-release nitrogen fertilizers. Therefore, the current research on optimizing fertilization methods mainly focuses on the combination of slow-release nitrogen fertilizer and urea^17,18^ to achieve the effect of cost saving and yield increase. Yi et al.^19^ found that the combined application of slow-release nitrogen fertilizer and urea could increase the yield and nitrogen accumulation of summer maize with different dosages of slow-release nitrogen fertilizer and urea. Ma et al.^20^ and Li et al.^21^ showed that the combination of slow-release nitrogen fertilizer and urea could significantly improve crop nitrogen partial productivity, apparent nitrogen use efficiency, agronomic efficiency and nitrogen harvest index, and increase yield.

Rational tillage practices can improve soil physical properties, improve crop growth conditions, and better meet crop demand, thereby increasing crop yields^22^. In recent years, with the popularization of conservation tillage, tillage techniques such as rotary tillage and no-tillage have been widely applied due to their simple operation and low energy consumption^23^. Conservation tillage is the process of improving soil health and further protecting soil by reducing the frequency and intensity of field preparation operations, thereby increasing crop yields. Rotary tillage and subsoiling are both commonly used conservation tillage techniques^24^. Studies have shown that long-term rotary tillage can cause problems such as shallow tillage layer and increased bulk density of deep soil^25^. The advantage of subsoiling is that it can break the bottom layer of the plough, improve soil permeability, enhance soil infiltration capacity, and thus improve soil water storage capacity and deep soil water content without turning over the soil layer, creating a good soil environment for crop growth, thereby enhancing the absorption capacity of roots to nutrients and water, and improving fertilizer absorption and utilization efficiency and yield^26–29^. Luo et al.^30^ and Zhang et al.^31^ showed that subsoiling could increase the dry weight and number of maize roots per plant, improve the activity of maize root plant protective enzymes, reduce the degree of cell membrane peroxidation, and slow the aging of maize roots, which was conducive to maintaining root vitality and improving the absorption of water and nutrients by roots in the late growth stage of maize. Wang et al.^32^ and Zhao et al.^33^ found that subsoiling can loosen the soil and promote the deep growth of maize roots, which is conducive to the absorption of water and nutrients by the roots and improves the yield and water use efficiency of maize grains. Yang et al.^34^ showed that compared with rotary tillage and subsoiling, deep rotary loosening tillage can improve soil hydrothermal conditions, increase crop dry matter accumulation, and then increase crop yield. *Entry point of this study* At present, there have been relevant studies on the effects of tillage methods and nitrogen fertilizer types on maize yield, nitrogen uptake and utilization at home and abroad, but there are few systematic studies on the effects of tillage and fertilization on soil moisture, dry matter accumulation, nitrogen uptake and utilization, operation and yield in maize fields in dryland tillage areas. On this basis, the effects of the combined application of slow-release nitrogen fertilizer and urea on soil water use efficiency, dry matter accumulation, nitrogen uptake and utilization, operation and maize yield in maize farmland were studied under two tillage methods, rotary tillage and rotary tillage + subsoiling in dry farming area, so as to screen out better tillage and fertilization methods in dry farming area, in order to provide a theoretical and scientific basis for selecting suitable tillage mode and optimal fertilization ratio in areas with similar ecological types.

## Materials and methods

### Overview of the test site

**Fig. 1:**
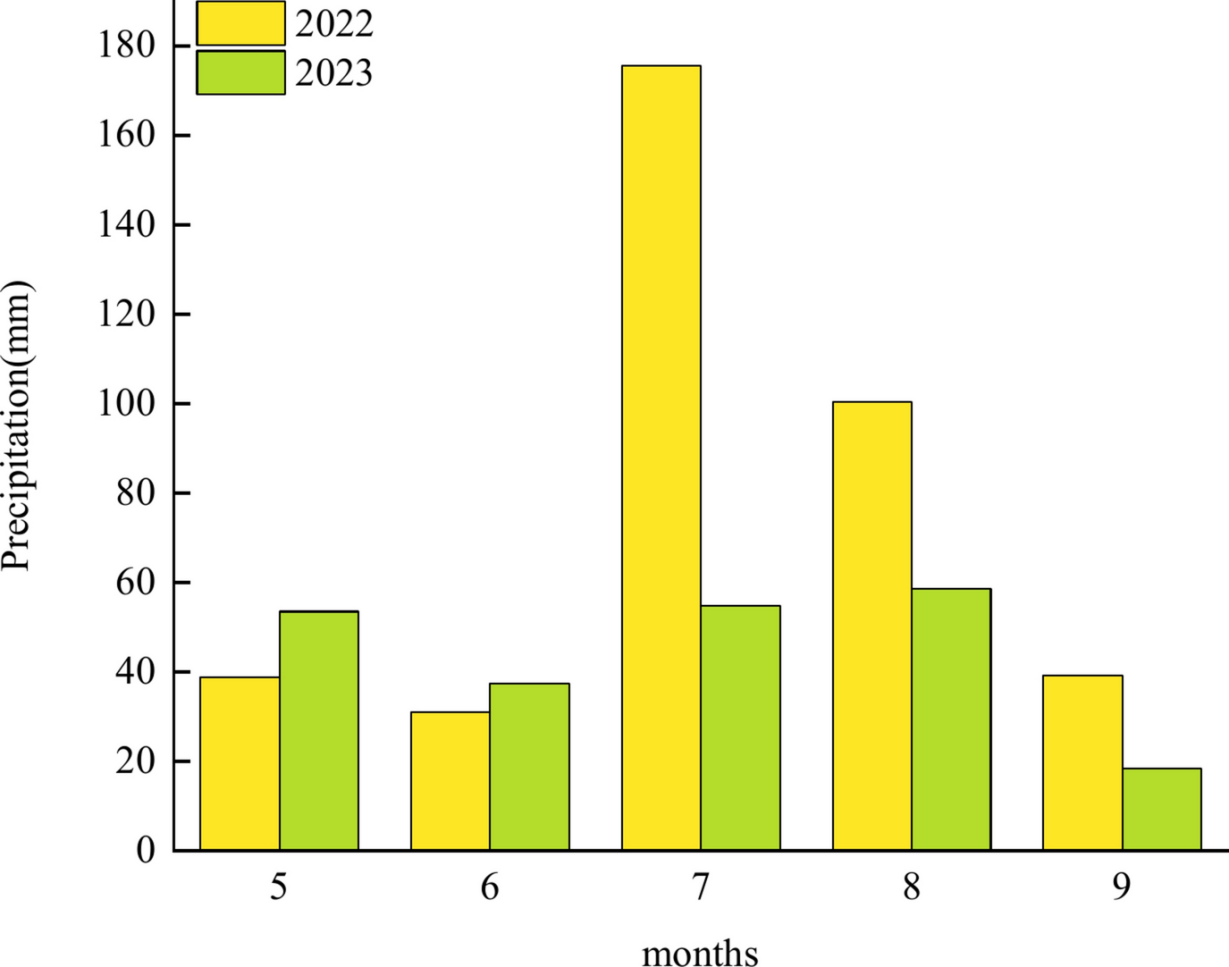
Precipitation the growth period of Dong yang Demonstration Base in 2022 and 2023.

The test site will be conducted in 2022–2023 at the Dongyang Experimental Base of Shanxi Agricultural University (Shanxi Academy of Agricultural Sciences) (37° 33′21″ N, 112° 40′2″ E, 802 m above sea level). The region belongs to the warm temperate semi-humid continental monsoon climate, with an average annual temperature of 9.7 °C, an annual average frost-free period of 158 days, rain and heat in the same season, and the rainfall is mainly concentrated in July and September, with 385 mm of precipitation in the growth period in 2022 and 222.9 mm in the growth period of 2023. The soil of the experimental plot was yellow clay, and the content of organic matter in the 0–20 cm tillage layer was 14.08 g kg^−1^, the total nitrogen content was 0.96 mg kg^−1^, the alkaline nitrogen content was 67.1 mg kg^−1^, the total phosphorus content was 0.775 mg kg^−1^, the available phosphorus content was 11.0 mg kg^−1^, the total potassium content was 17.06 g kg^−1^, the available potassium content was 110 mg kg^−1^, and the pH value was 8.55. The rainfall during the growing season in 2022 and 2023 is shown in Fig. 1.

### Test materials

(1) Seeds: Drought-resistant maize variety Qiangsheng 388 is selected, with compact plants, 317 cm high, 116 cm ears, 22 leaves, and a growth period of 129 days. The seedling leaf sheath is dark purple, the leaf margin is red, the filament is green, medium resistance to large spot disease, and high resistance to stem rot. Provided by Shanxi Qiangsheng Seed Industry Co., Ltd. (2) Nitrogen fertilizer: urea, net content of 40 kg, total nitrogen content ≥ 46.0%, particle size range of 0.85 mm ~ 2.80 mm ≥ 90%, provided by PetroChina Co., Ltd. slow-release nitrogen fertilizer: Jugu Neng brand was selected, with a net content of 40 kg, a total nutrient content of ≥ 50% (the mass ratio of N:P_2_O_5_:K_2_O was 26:12:12), and a slow-release nitrogen ≥ 8%. Provided by Jiashili (Yingcheng) Fertilizer Co., Ltd.

### Experimental design

The two-factor randomized block design was adopted, and the tillage methods were rotary tillage (R) and rotary tillage + subsoiling (R + S), in which the depth of rotary tillage reached 30 cm and the depth of subsoiling reached 50 cm. The fertilization methods were CK (no fertilization treatment), 100% urea (U), 100% slow-release nitrogen fertilizer (S), and combined application of urea and slow-release nitrogen fertilizer (UNS), with the ratios of 2:8 (UNS1), 3:7 (UNS2) and 4:6 (UNS3), respectively. The planting density was 66,000 plants hm^−2^, the plot area was 60 m^2^, and each treatment was set up for 3 replicates.The specific tillage design and fertilization amount are shown in Table 1.

**Table 1:** Presentation of tillage practices and fertilizer application design.

Farming methods	Fertilizer treatment	nitrogenous fertilizer	slow-release nitrogen fertilizer
R	CK	0 kg hm^−2^ urea	0 kg hm^−2^ slow-release nitrogen fertilizer
	U	300 kg hm^−2^ urea	0 kg hm^−2^ slow-release nitrogen fertilizer
	S	0 kg hm^−2^ urea	530.7 kg hm^−2^ slow-release nitrogen fertilizer
	UNS1	60 kg hm^−2^ urea	424.6 kg hm^−2^ slow-release nitrogen fertilizer
	UNS2	90 kg hm^−2^ urea	371.5 kg hm^−2^ slow-release nitrogen fertilizer
	UNS3	120 kg hm^−2^ urea	318.4 kg hm^−2^ slow-release nitrogen fertilizer
R + S	CK	0 kg hm^−2^ urea	0 kg hm^−2^ slow-release nitrogen fertilizer
	U	300 kg hm^−2^ urea	0 kg hm^−2^ slow-release nitrogen fertilizer
	S	0 kg hm^−2^ urea	530.7 kg hm^−2^ slow-release nitrogen fertilizer
	UNS1	60 kg hm^−2^ urea	424.6 kg hm^−2^ slow-release nitrogen fertilizer
	UNS2	90 kg hm^−2^ urea	371.5 kg hm^−2^ slow-release nitrogen fertilizer
	UNS3	120 kg hm^−2^ urea	318.4 kg hm^−2^ slow-release nitrogen fertilizer

R stands for rotary tillage; R + S means rotary tillage + subsoiling; CK means no fertilization; U means 100% nitrogen fertilizer, i.e., 300 kg hm^−2^ urea; S indicates 100% application of slow-release nitrogen fertilizer, i.e., 530.7 kg hm^−2^; UNS indicates the combined application of urea and slow-release nitrogen fertilizer, which is divided into three ratios: (1) the ratio of urea to slow-release nitrogen fertilizer is 2:8, i.e., 60 kg hm^−2^ urea and 424.6 kg hm^−2^ slow-release nitrogen fertilizer, (2) the ratio of urea to slow-release nitrogen fertilizer is 3:7, i.e., 90 kg hm^−2^ urea and 371.5 kg hm^−2^ slow-release nitrogen fertilizer, and (3) the ratio of urea to slow-release nitrogen fertilizer is 4:6, i.e., 120 kg hm^−2^ urea and 318.4 kg hm^−2^ slow-release nitrogen fertilizer.

### Determination items and methods

#### Soil moisture

Before sowing and after harvesting, each test plot was sampled in the 0–100 cm soil layer with a ring knife with a volume of 100 cm^3^, and every 20 cm was a layer, repeated three times. The soil moisture content and soil bulk density were determined by drying method, so as to calculate the water consumption and water use efficiency during the growth period.

$$\begin{aligned} \theta &= \, \left( {{\text{fresh soil sample }} - {\text{ dry soil sample}}} \right)/{\text{dry soil sample}}*{1}00\% \\ {\text{R }} &= {\text{ dry soil sample}}/{1}00 \\ {\text{W}} &= \theta *{\text{h}}*{\text{R}}*{1}0 \\ {\text{ETa}} &= \left( {{\text{W}}_{{1}} - {\text{W}}_{{2}} } \right) + {\text{P}} \\ {\text{WUE}}\left( {{\text{kg}}\;{\text{hm}}^{{ - {2}}} \;{\text{mm}}^{{ - {1}}} } \right) &= {\text{Y}}/{\text{ETa}} \\ \end{aligned}$$

wherein: θ is the soil moisture content (%), R is the soil bulk density (g cm^-3^), h is the soil depth (cm), and 10 is the unit conversion factor. W is the soil water storage (mm), W_1_ is the soil water storage before sowing (mm), and W_2_ is the soil water storage after harvest (mm). ETa is the water consumption of crops (mm); P is the rainfall during the growth period (mm). WUE is water use efficiency (kg hm^−2^ mm^−1^); Y is the crop yield (kg hm^−2^)

#### Aboveground dry matter quality and total nitrogen in aboveground parts of plants

Sampling was carried out at the jointing stage, the large flare stage, the tasseling stage, the grain filling stage and the maturity stage, and 3 maize plants were selected for each plot at one time and repeated 3 times. Among them, the jointing stage, the large flare stage and the tasseling stage were divided into two parts according to the stem and leaves, and the grain filling stage and the mature stage were divided into four parts according to the stem, leaf, leaf wrapping + cob and grain. Samples were placed in an oven at 105 °C for 0.5 h and then at 75 °C to constant weight, recording the dry matter mass. The dried samples were sieved at 0.5 mm, boiled with H_2_SO_4_-H_2_O_2_, and analyzed for total nitrogen content by Kjeldahl method.

Formula:

- Accumulation of dry matter weight of plants = dry matter weight per plant × number of plants per unit area
- Plant nitrogen accumulation (kg hm^−2^) = plant nitrogen content (%) × dry matter mass × hectare density
- Nitrogen uptake efficiency (kg kg^−1^) = nitrogen accumulation/nitrogen application
- Nitrogen use efficiency (kg kg^−1^) = grain yield/plant nitrogen accumulation
- Nitrogen fertilizer use efficiency (kg kg^−1^) = grain yield/nitrogen application rate
- Nitrogen harvest index (%) = total nitrogen accumulation in grains/total nitrogen accumulation in plants × 100
- Nitrogen transfer of vegetative organs = nitrogen uptake of vegetative organs at the tasseling stage − nitrogen uptake by vegetative organs at maturity stage
- Nitrogen uptake after tasseling stage = total nitrogen uptake at mature stage − nitrogen uptake by vegetative organs at tasseling stage
- Nitrogen transfer rate of vegetative organs = nitrogen transfer of vegetative organs/nitrogen uptake of vegetative organs at the tasseling stage × 100%

#### Yield

The middle 2 rows of each plot were taken to measure the yield, and the total number of plants and the actual number of harvested ears were recorded. At the same time, 20 fruit ears were randomly selected to be naturally air-dried, and when the moisture was less than 20%, the panicle length (cm), bald tip length (cm) and grain number per spike were measured, and the 100-grain weight (g) was determined after threshing and air drying, and the grain yield was calculated according to the grain moisture content of 14% by combining the seed test data with the field yield measurement data.

### Data processing and analysis

Use Microsoft Excel 2023 software for data collation; SPSS 22.0 statistical analysis software was used to analyze the significance of the test data, and the LSD method was used to compare the test data under the condition of 5% significance, and the difference was significant when the *P* < 0.05 was used. The data results are represented as “mean ± standard error”, and the data is graphed using Origin2021.

## Results and analysis

### Dry matter accumulation

**Fig. 2:**
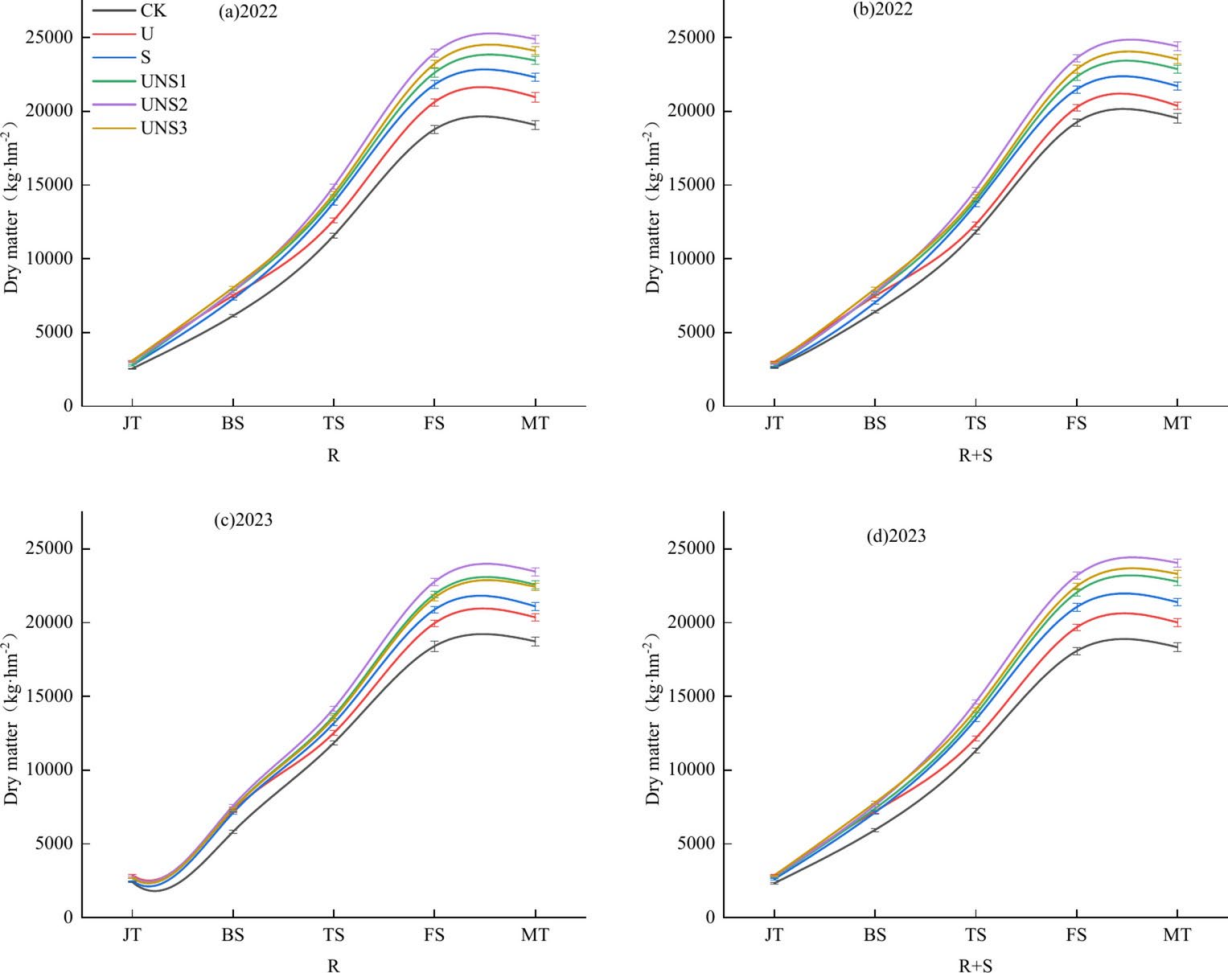
Effects of different tillage and fertilizer types on dry accumulation in maize plants in 2022 and 2023. *Note* R: rotary tillage; R + S: rotary tillage + subsoiling; CK: no fertilization; U: 300 kg hm^−2^ urea, 0 kg hm^−2^ slow-release nitrogen fertilizer; S: 0 kg hm^−2^ urea, 530.7 kg hm^−2^ slow-release nitrogen fertilizer; UNS1: 60 kg hm^−2^ urea, 424.6 kg hm^−2^ slow-release nitrogen fertilizer; UNS2: 90 kg hm^−2^ urea, 371.5 kg hm^−2^ slow-release nitrogen fertilizer; UNS3: 120 kg hm^−2^ urea, 318.4 kg hm^−2^ slow-release nitrogen fertilizer; JT: jointing stage; BS: Big trumpet stage; TS: Tasseling stage; FS: Filling stage; MT: maturity stage;

As can be seen from Fig. 2, the dry matter accumulation of all treatments gradually increased with the advancement of maize growth period. At the jointing stage, the higher the proportion of urea in the treatment, the greater the dry matter accumulation, in the order of U > UNS3 > UNS2 > UNS1 > S > CK. With the advancement of the growth period, the effect of slow-release nitrogen fertilizer on dry matter accumulation was significant, and the order was UNS > S > U > CK.

Under the same tillage method, the dry matter accumulation in the nitrogen application treatment was significantly higher than that in the non-nitrogen application treatment. At the maturity stage, UNS2 was the best under R and R + S tillage methods in 2022, which were 24,898.5 kg hm^−2^ and 24,409.44 kg hm^−2^, respectively. CK was the lowest, with R CK and R + S CK being 19,074.66 kg hm^−2^ and 19,534.02 kg hm^−2^, respectively. The treatment of R UNS2 was 2.00% higher than that of R + S UNS2.

In 2023, UNS2 was the best treatment under the two tillage methods, with R UNS2 and R + S UNS2 being 23,464.98 kg hm^−2^ and 24,056.34 kg hm^−2^, respectively, which were 25.24% and 31.17% higher than those of R CK and R + S CK, respectively. R + S UNS2 treatment was 2.52% higher than that of R UNS2.

### Nitrogen accumulation

**Fig. 3:**
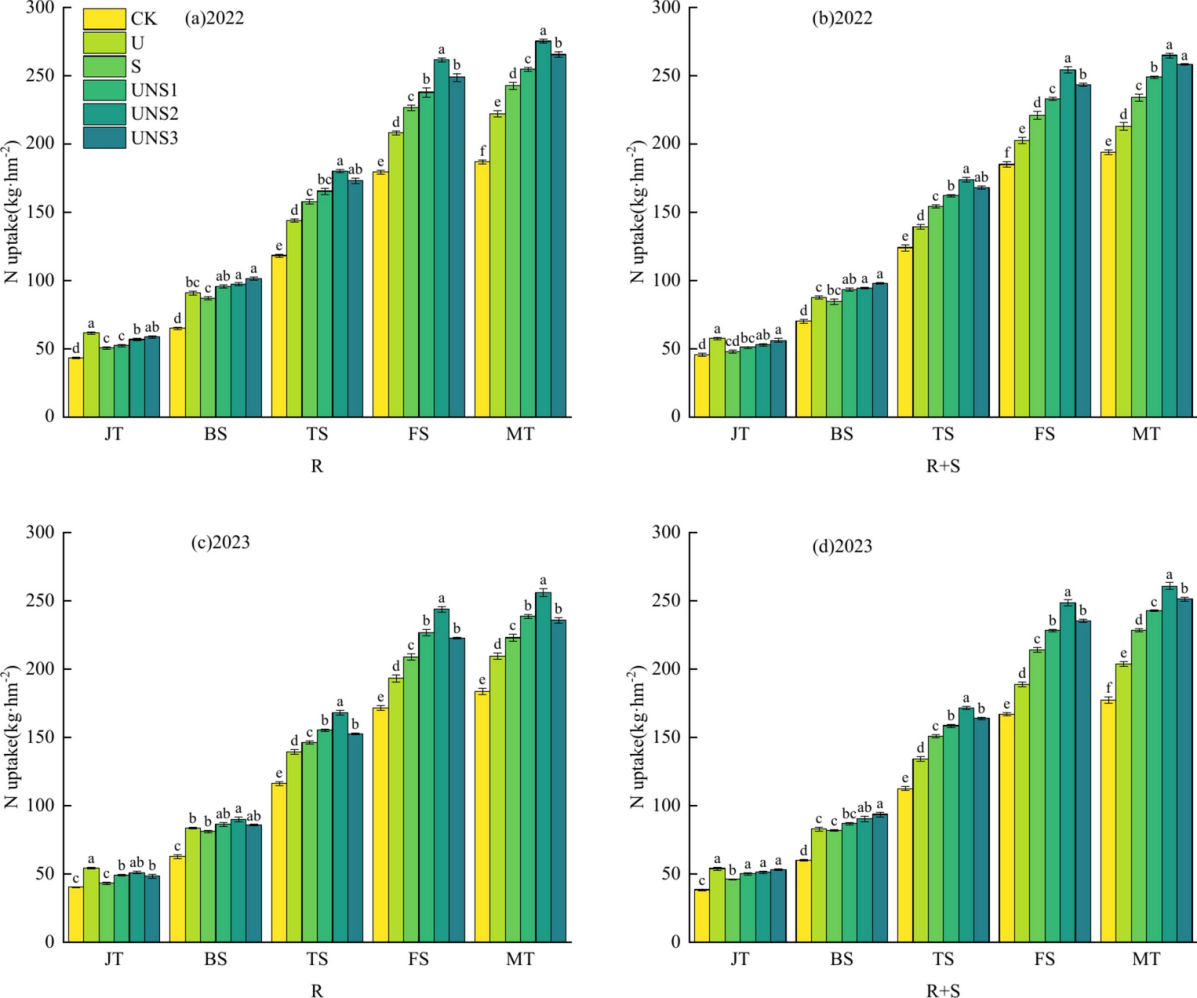
Effects of different tillage and fertilizer types on nitrogen accumulation in maize plants in 2022 and 2023. Note: R: rotary tillage; R + S: rotary tillage + subsoiling; CK: no fertilization; U: 300 kg hm^−2^ urea, 0 kg hm^−2^ slow-release nitrogen fertilizer; S: 0 kg hm^−2^ urea, 530.7 kg hm^−2^ slow-release nitrogen fertilizer; UNS1: 60 kg hm^−2^ urea, 424.6 kg hm^−2^ slow-release nitrogen fertilizer; UNS2: 90 kg hm^−2^ urea, 371.5 kg hm^−2^ slow-release nitrogen fertilizer; UNS3: 120 kg hm^−2^ urea, 318.4 kg hm^−2^ slow-release nitrogen fertilizer; JT: jointing stage; BS: Big trumpet stage; TS: Tasseling stage; FS: Filling stage; MT: maturity stage; a, b, c, d represent different levels of significance. a indicates a higher level of significance, such as 0.01 or 0.05, b may indicate a secondary level of significance, and c may indicate a lower level of significance.

It can be seen from Fig. 3 that with the advancement of maize growth period, the nitrogen accumulation of all treatments were consistent with the previous dry matter accumulation, and showed a gradual increasing trend. At the jointing stage, the higher the proportion of urea in the treatment, the greater the nitrogen accumulation, in the order of U > UNS3 > UNS2 > UNS1 > S > CK. With the advancement of the growth period, the effect of slow-release nitrogen fertilizer on nitrogen accumulation was significant, and the order was UNS > S > U > CK.

Under the same tillage method, the nitrogen accumulation in the nitrogen application treatment was significantly higher than that in the non-nitrogen application treatment. At the maturity stage, the nitrogen accumulation of UNS2 treatment reached the maximum under the two tillage methods of R and R + S in 2022, which were 275.25 kg hm^−2^ and 264.88 kg hm^−2^, respectively. The CK treatment was the lowest, with R CK and R + S CK being 186.85 kg hm^−2^ and 194.02 kg hm^−2^, respectively. The treatment of R UNS2 was 3.88% higher than that of R + S UNS2. In 2023, the nitrogen accumulation of the two tillage methods UNS2 was also the best, with R UNS2 and R + S UNS2 being 256.08 kg hm^−2^ and 260.81 kg hm^−2^, respectively, which were 39.43% and 47.09% higher than those of R CK and R + S CK treatments, respectively. R + S UNS2 treatment was 1.85% higher than that of R UNS2.

### Output and production components

It can be seen from Table 2 that the yield of maize under different tillage methods was UNS > S > U > CK in the two-year experiment, and UNS2 had the highest yield. In 2022, the yield of UNS2 treatment was 14,712.78 kg hm^−2^, which was 4.15% ~ 56.85% higher than that of the other five treatments. The maximum mass of 100 grains in UNS2 treatment was 44.46 g, which was 3.68% ~ 19.36% higher than that of the other five treatments. The yield of UNS2 treatment was 14,138.3 kg hm^−2^, which was 2.93% ~ 43.37% higher than that of the other five treatments. The mass of 100 grains in UNS2 treatment was 44.13 g, which was 4.76% ~ 16.10% higher than that of the other 5 treatments. The bald tip length of the two tillage treatments were significantly lower than that of the other treatments, and the average spike length and grain number per spike were significantly better than those of the other treatments. The yield and 100-grain weight of R UNS2 treatment were 4.06% and 0.75% higher than those of R + S UNS2, respectively.

**Table 2:** Effects of different tillage and fertilizer types on yields and its components of maize.

Year	Treatment	Ear length (cm)	Bald ear length (cm)	Kernel number ear row	hundred- grain weight(g)	Yield (kg hm^−2^)
2022	R-CK	17.42 ± 0.19e	1.29 ± 0.06a	538.58 ± 7.32d	37.25 ± 0.24e	9380.39 ± 120.44f.
	R-U	18.96 ± 0.15d	1.17 ± 0.06ab	571.11 ± 8.58c	39.63 ± 0.29d	11,579.21 ± 176.06e
	R-S	19.35 ± 0.13 cd	1.03 ± 0.08bc	593.68 ± 5.25c	40.63 ± 0.26c	12,834.44 ± 191.84d
	R-UNS1	19.7 ± 0.14bc	0.85 ± 0.04 cd	594.83 ± 5.4c	41.96 ± 0.32b	13,515.26 ± 143.06c
	R-UNS2	20.86 ± 0.17a	0.47 ± 0.06e	647.95 ± 8.58a	44.46 ± 0.31a	14,712.78 ± 125.73a
	R-UNS3	20.07 ± 0.2b	0.68 ± 0.03d	621.56 ± 6.57b	42.88 ± 0.25b	14,126.07 ± 149.22b
	R + S-CK	17.81 ± 0.13e	1.26 ± 0.07a	542.16 ± 10.52d	38.01 ± 0.35e	9861.2 ± 106.55e
	R + S-U	18.75 ± 0.09d	1.21 ± 0.05a	567.03 ± 5.65 cd	39.38 ± 0.31d	11,084.3 ± 153.95d
	R + S–S	19.23 ± 0.12 cd	1.12 ± 0.03ab	575.98 ± 7.94bc	40.5 ± 0.37c	12,329.21 ± 196.31c
	R + S-UNS1	19.57 ± 0.16bc	0.97 ± 0.06bc	588.91 ± 8.89bc	41.23 ± 0.22bc	13,198.07 ± 155.42b
	R + S-UNS2	20.6 ± 0.18a	0.61 ± 0.03d	635.09 ± 9.5a	44.13 ± 0.12a	14,138.3 ± 146.59a
	R + S-UNS3	20.02 ± 0.14b	0.82 ± 0.04c	603.66 ± 7.79b	42.13 ± 0.27b	13,735.86 ± 134.01a
Significant level(*F* value)						
Tillage(T)		ns	*	ns	ns	**
Fertilization(F)		**	**	**	**	**
T × F		ns	ns	ns	ns	*
2023	R-CK	17.34 ± 0.08d	1.68 ± 0.09a	527.96 ± 8.6d	35.88 ± 0.33d	9201.24 ± 121.29e
	R-U	18.34 ± 0.17c	1.47 ± 0.07b	548.28 ± 5.85 cd	39.23 ± 0.22c	10,892.03 ± 144.98d
	R-S	18.775 ± 0.08c	1.41 ± 0.04bc	556.02 ± 6.44bcd	40.12 ± 0.28bc	11,708.27 ± 174.17c
	R-UNS1	19.4 ± 0.14b	1.21 ± 0.05bc	580.62 ± 8.4b	41.03 ± 0.27b	12,637.85 ± 210.8b
	R-UNS2	20.1 ± 0.16a	0.87 ± 0.03d	617.19 ± 10.42a	43.33 ± 0.31a	13,602.98 ± 161.93a
	R-UNS3	19.28 ± 0.19b	1.25 ± 0.06c	571.26 ± 8.35bc	40.88 ± 0.35b	12,429.2 ± 198.65b
	R + S-CK	17.36 ± 0.105e	1.73 ± 0.12a	521.84 ± 4.39d	35.49 ± 0.36e	9101.04 ± 109.89e
	R + S-U	18.08 ± 0.07d	1.48 ± 0.1ab	542.31 ± 7.2d	39.03 ± 0.32d	10,588.89 ± 158.94d
	R + S–S	19.04 ± 0.195c	1.26 ± 0.09bc	572 ± 6.9c	40.24 ± 0.21c	12,030.2 ± 188.75c
	R + S-UNS1	19.49 ± 0.16c	1.14 ± 0.03c	585.22 ± 7.22bc	41.14 ± 0.31bc	12,849.21 ± 113.16b
	R + S-UNS2	20.46 ± 0.11a	0.75 ± 0.04d	625.9 ± 9.83a	43.58 ± 0.42a	13,901.88 ± 154.68a
	R + S-UNS3	19.96 ± 0.12b	1.03 ± 0.04c	598.24 ± 7.32b	42.01 ± 0.33b	13,347.81 ± 128.61b
Significant level(*F* value)						
Tillage(T)		*	*	ns	ns	*
Fertilization(F)		**	**	**	**	**
T × F		ns	ns	ns	ns	*

R: rotary tillage; R + S: rotary tillage + subsoiling; CK: no fertilization; U: 300 kg hm^−2^ urea, 0 kg hm^−2^ slow-release nitrogen fertilizer; S: 0 kg hm^−2^ urea, 530.7 kg hm^−2^ slow-release nitrogen fertilizer; UNS1: 60 kg hm^−2^ urea, 424.6 kg hm^−2^ slow-release nitrogen fertilizer; UNS2: 90 kg hm^−2^urea, 371.5 kg hm^−2^ slow-release nitrogen fertilizer; UNS3: 120 kg hm^−2^ urea, 318.4 kg hm^−2^ slow-release nitrogen fertilizer; Different alphabets indicate the significance within the same year at 5% level by LSD test. ns, not significant, (*p * > 0.05).* Significant at *p * < 0.05. ** Significant at p < 0.01.

In 2023, the yield of UNS2 treatment was the best under R tillage mode, which was 13,602.98 kg hm^−2^, followed by UNS1 treatment, which was 12,637.85 kg hm^−2^, and CK was the lowest, which was 9201.24 kg hm^−2^. The yield of UNS2 treatment was 13,901.88 kg hm^−2^ under R + S tillage mode, followed by UNS3 treatment at 13,347.81 kg hm^−2^. The lowest CK was 9101.04 kg hm^−2^. The 100-grain mass R and R + S tillage methods were the highest in UNS2, which were 43.33 g and 43.58 g, respectively, which corresponded with the yield. The yield and 100-grain weight of R + S UNS2 treatment were 2.20% and 0.58% higher than those of R UNS2, respectively.

### Nitrogen uptake and utilization

As shown in Table 3, the nitrogen uptake and utilization of maize plants under the two tillage methods showed UNS > S > U > CK under the two tillage methods, and UNS2 was the highest.In 2022, R UNS2 and R + S UNS2 had the highest nitrogen absorption efficiency, which were 0.92 kg kg^−1^ and 0.88 kg kg^−1^, respectively. U was the lowest, and R U and R + S U were 0.74 kg kg^−1^ and 0.71 kg kg^−1^, respectively. The nitrogen harvest index R UNS2 and R + S UNS2 were the highest, which were 64.61% and 63.74%, respectively. CK was the smallest, with 56.3% and 60.77% of R CK and R + S CK, respectively. The nitrogen uptake efficiency and nitrogen harvest index of R UNS2 treatment were 4.55% and 1.36% higher than those of R + S UNS2, respectively. The nitrogen use efficiency and nitrogen fertilizer use efficiency of the two tillage methods were significantly better than those of the other treatments.

**Table 3:** Effects of different tillage and fertilizer types on nitrogen uptake and use efficiency of maize.

Year	Treatment	N uptake efficiency (kg kg^−1^)	N use efficiency (kg kg^−1^)	N productive efficiency (kg kg^−1^)	N harvest Index (%)
2022	R-CK	0	50.2 ± 0.02e	0	56.3 ± 0.08e
	R-U	0.74 ± 0.02c	52.11 ± 0.03d	38.6 ± 0.35e	61.49 ± 0.26d
	R-S	0.81 ± 0.03bc	52.92 ± 0.05c	42.78 ± 0.43d	62.54 ± 0.21c
	R-UNS1	0.85 ± 0.02ab	53.08 ± 0.07bc	45.05 ± 0.54c	63.59 ± 0.18b
	R-UNS2	0.92 ± 0.01a	53.45 ± 0.06a	49.04 ± 0.27a	64.61 ± 0.17a
	R-UNS3	0.88 ± 0.01ab	53.22 ± 0.07b	47.09 ± 0.2b	63.84 ± 0.13b
	R + S-CK	0	50.82 ± 0.05e	0	57.7 ± 0.37d
	R + S-U	0.71 ± 0.03c	52.09 ± 0.06d	36.95 ± 0.23e	60.77 ± 0.21c
	R + S–S	0.78 ± 0.03bc	52.72 ± 0.07c	41.1 ± 0.27d	61.64 ± 0.31b
	R + S-UNS1	0.83 ± 0.02ab	53.03 ± 0.09b	43.99 ± 0.26c	63.09 ± 0.2a
	R + S-UNS2	0.88 ± 0.01a	53.38 ± 0.04a	47.13 ± 0.55a	63.74 ± 0.16a
	R + S-UNS3	0.86 ± 0.02a	53.19 ± 0.07ab	45.79 ± 0.31b	63.46 ± 0.15a
Significant level(*F* value)					
Tillage(T)		ns	ns	**	**
Fertilization(F)		**	**	**	**
T × F		ns	ns	ns	ns
2023	R-CK	0	50.1 ± 0.04e	0	56.2 ± 0.22e
	R-U	0.7 ± 0.02c	51.99 ± 0.03d	36.31 ± 0.18e	59.96 ± 0.21d
	R-S	0.74 ± 0.03bc	52.52 ± 0.09c	39.03 ± 0.28c	61.31 ± 0.18c
	R-UNS1	0.8 ± 0.01ab	52.92 ± 0.05ab	42.13 ± 0.25b	62.73 ± 0.19b
	R-UNS2	0.85 ± 0.01a	53.12 ± 0.03a	45.34 ± 0.27a	63.43 ± 0.14a
	R-UNS3	0.79 ± 0.02b	52.75 ± 0.09b	41.43 ± 0.5b	62.57 ± 0.21b
	R + S-CK	0	51.33 ± 0.06e	0	55.52 ± 0.33e
	R + S-U	0.68 ± 0.01c	51.91 ± 0.05d	35.3 ± 0.28d	59.48 ± 0.31d
	R + S–S	0.76 ± 0.03b	52.64 ± 0.06c	41.1 ± 0.31c	61.45 ± 0.12c
	R + S-UNS1	0.81 ± 0.02ab	52.95 ± 0.08b	42.83 ± 0.71bc	62.81 ± 0.08b
	R + S-UNS2	0.87 ± 0.01a	53.3 ± 0.03a	46.34 ± 0.32a	63.59 ± 0.08a
	R + S-UNS3	0.84 ± 0.02a	53.13 ± 0.07ab	44.49 ± 0.65b	63.25 ± 0.13ab
Significant level(*F* value)					
Tillage(T)		ns	**	**	ns
Fertilization(F)		**	**	**	**
T × F		ns	*	**	ns

R: rotary tillage; R + S: rotary tillage + subsoiling; CK: no fertilization; U: 300 kg hm^−2^urea, 0 kg hm^−2^ slow-release nitrogen fertilizer; S: 0 kg hm^−2^ urea, 530.7 kg hm^−2^ slow-release nitrogen fertilizer; UNS1: 60 kg hm^−2^ urea, 424.6 kg hm^−2^ slow-release nitrogen fertilizer; UNS2: 90 kg hm^−2^ urea, 371.5 kg hm^−2^ slow-release nitrogen fertilizer; UNS3: 120 kg hm^−2^ urea, 318.4 kg hm^−2^ slow-release nitrogen fertilizer; Different alphabets indicate the significance within the same year at 5% level by LSD test. ns, not significant, (*p * > 0.05).* Significant at *p * < 0.05. ** Significant at p < 0.01.

In 2023, the nitrogen uptake efficiency of R and R + S tillage methods was the same as that in 2022, and the UNS2 treatment was the highest, which was 0.85 kg kg^−1^ and 0.87 kg kg^−1^, respectively. U was the lowest, with R U and R + S U being 0.70 kg kg^−1^ and 0.68 kg kg^−1^, respectively. The nitrogen harvest index UNS2 was the first treatment, with R UNS2 and R + S UNS2 reaching 63.43% and 63.59%, respectively. CK was the smallest, with 56.2% and 55.52% of R’CK and R + S CK, respectively. The nitrogen uptake efficiency and nitrogen harvest index of R + S UNS2 treatment were 2.35% and 0.25% higher than those of RUNS2, respectively.

### Nitrogen transport

It can be seen from Table 4 that in all the treatments of the two-year experiment, the nitrogen uptake stored in the vegetative organs of maize plants at the tasseling stage was greater than that at the maturity stage, which indicated that the nitrogen stored in the vegetative organs was transferred to the grain after the tasseling stage, and it showed a trend of UNS > S > U > CK, in which the UNS2 treatment reached the maximum. In 2022, the nitrogen turnover of UNS2 treatment was 82.64 kg hm^−2^, which was 7.33–126.03% higher than that of the other five treatments. The nitrogen operation efficiency of UNS2 treatment was 45.9%, which was 3.12–48.39% higher than that of the other five treatments. R + S tillage method the nitrogen flux of UNS2 treatment was 77.83 kg hm^−2^, which was 5.53–85.70% higher than that of the other five treatments. The nitrogen operation efficiency of UNS2 treatment was 44.76%, which was 2.02–45.75% higher than that of the other five treatments.

**Table 4:** Effects of different tillage methods and fertilization types on nitrogen accumulation and operation of maize before and after tasseling.

Year	Treatment	Nitrogen content in vegetative organs at the tasseling stage (kg hm^−2^)	Nitrogen content of mature plants(kg hm^−2^)		Nitrogen uptake after tasseling (kg hm^−2^)	Nitrogen transport in vegetative organs (kg hm^−2^)	Nitrogen transport efficiency (%)
			Vegetative	Total			
2022	R-CK	118.19 ± 1.43e	81.63 ± 0.55e	186.85 ± 2.28f.	68.66 ± 1.57e	36.56 ± 0.79f.	30.93 ± 0.52e
	R-U	144.01 ± 1.81d	85.55 ± 0.62d	222.19 ± 3.05e	78.18 ± 1.16d	58.45 ± 1.39e	40.59 ± 0.63d
	R-S	157.58 ± 2.34c	90.87 ± 0.81c	242.54 ± 3.33d	84.97 ± 0.74c	66.71 ± 1.18d	42.34 ± 0.2c
	R-UNS1	165.37 ± 3.33bc	92.7 ± 1.09bc	254.61 ± 1.89c	89.24 ± 0.78b	72.66 ± 1.2c	43.94 ± 0.23b
	R-UNS2	180.05 ± 1.47a	97.41 ± 1.25a	275.25 ± 2.21a	95.2 ± 0.94a	82.64 ± 0.71a	45.9 ± 0.18a
	R-UNS3	172.97 ± 2.65ab	95.98 ± 1.17ab	265.42 ± 3.06b	92.45 ± 0.57ab	76.99 ± 0.76b	44.51 ± 0.2b
	R + S-CK	123.99 ± 3.2e	82.08 ± 0.7d	194.02 ± 2.51e	70.04 ± 1.09e	41.91 ± 1.23e	33.8 ± 0.76e
	R + S-U	139.28 ± 2.39d	83.47 ± 0.81d	212.78 ± 3.87d	73.5 ± 0.69d	55.81 ± 1.95d	40.07 ± 0.21d
	R + S–S	154.37 ± 1.85c	89.71 ± 1.02c	233.86 ± 3.51c	79.49 ± 0.47c	64.65 ± 1.32c	41.88 ± 0.24c
	R + S-UNS1	162.04 ± 1.23b	91.86 ± 0.92bc	248.88 ± 0.9b	86.84 ± 0.90b	70.18 ± 0.89b	43.31 ± 0.18b
	R + S-UNS2	173.87 ± 2.2a	96.04 ± 1.55a	264.88 ± 2.55a	91.01 ± 0.94a	77.83 ± 1.5a	44.76 ± 0.17a
	R + S-UNS3	168.13 ± 1.74ab	94.38 ± 1.12ab	258.26 ± 0.59a	90.13 ± 0.70a	73.75 ± 0.73ab	43.87 ± 0.15ab
Significant level(*F* value)							
Tillage(T)		ns	ns	*	**	*	ns
Fertilization(F)		**	**	**	**	**	**
T × F		ns	ns	ns	*	*	**
2023	R-CK	116.09 ± 1.93e	80.44 ± 0.73d	183.66 ± 3.1e	67.57 ± 0.54d	35.65 ± 1.59e	30.71 ± 0.45d
	R-U	139.41 ± 2.15d	83.88 ± 0.89 cd	209.52 ± 3.39d	70.1 ± 1.14d	55.53 ± 1.41d	39.83 ± 0.29c
	R-S	146.12 ± 1.55c	86.25 ± 1.04bc	222.94 ± 3.77c	76.81 ± 1.48c	59.87 ± 0.84 cd	40.97 ± 0.34c
	R-UNS1	155.19 ± 1.37b	88.7 ± 1.09b	238.81 ± 2.03b	83.62 ± 0.51b	66.19 ± 0.6b	42.65 ± 0.22b
	R-UNS2	168.22 ± 2.67a	93.64 ± 1.61a	256.08 ± 3.74a	87.86 ± 0.60a	74.58 ± 2.09a	44.33 ± 0.35a
	R-UNS3	152.56 ± 0.89b	88.2 ± 0.89b	235.64 ± 2.88b	83.08 ± 1.07b	64.36 ± 1.18bc	42.19 ± 0.3b
	R + S-CK	112.57 ± 2.22e	78.86 ± 0.77c	177.31 ± 3.22f.	64.74 ± 1.4e	33.71 ± 1.83e	29.94 ± 0.4e
	R + S-U	134.38 ± 2.45d	82.66 ± 1.1c	203.97 ± 2.44e	69.59 ± 1.33d	51.72 ± 1.27d	38.49 ± 0.16d
	R + S–S	151 ± 1.33c	88.41 ± 0.94b	228.55 ± 1.77d	77.55 ± 1.12c	62.9 ± 1.41c	41.65 ± 0.23c
	R + S-UNS1	158.52 ± 1.55b	90.64 ± 1.32b	242.67 ± 0.84c	84.15 ± 0.66b	68.28 ± 0.59b	43.07 ± 0.16b
	R + S-UNS2	171.49 ± 1.74a	94.97 ± 1.59a	260.81 ± 3.55a	89.32 ± 0.31a	76.52 ± 1.19a	44.62 ± 0.19a
	R + S-UNS3	163.99 ± 1.35b	92.32 ± 1.18ab	251.21 ± 1.82b	87.21 ± 0.44ab	71.67 ± 0.78b	43.7 ± 0.11b
Significant level(*F* value)							
Tillage(T)		*	ns	ns	ns	ns	ns
Fertilization(F)		**	**	**	**	**	**
T × F		**	ns	*	ns	*	**

R: rotary tillage; R + S: rotary tillage + subsoiling; CK: no fertilization; U: 300 kg hm^−2^urea, 0 kg hm^−2^ slow-release nitrogen fertilizer; S: 0 kg hm^−2^ urea, 530.7 kg hm^−2^ slow-release nitrogen fertilizer; UNS1: 60 kg hm^−2^ urea, 424.6 kg hm^−2^ slow-release nitrogen fertilizer; UNS2: 90 kg hm^−2^ urea, 371.5 kg hm^−2^ slow-release nitrogen fertilizer; UNS3: 120 kg hm^−2^ urea, 318.4 kg hm^−2^ slow-release nitrogen fertilizer; Different alphabets indicate the significance within the same year at 5% level by LSD test. ns, not significant, (*p * > 0.05).* Significant at *p * < 0.05. ** Significant at p < 0.01.

In 2023, the nitrogen transmutation UNS2 treatment was the maximum under R and R + S tillage methods, and the R UNS2 and R + S UNS2 were 74.58 kg hm^−2^ and 76.52 kg hm^−2^, respectively. CK was the smallest, with 35.65 kg hm^−2^ and 33.71 kg hm^−2^, respectively. R + S UNS2 treatment was 2.60% higher than R UNS2 treatment. The nitrogen operation efficiency was the highest under the two tillage methods, and the R UNS2 and R + S UNS2 were 44.33% and 44.62%, respectively. CK was the lowest, at 30.71% and 29.94%, respectively. R + S UNS2 treatment was 0.65% higher than R UNS2 treatment.

### Water use

Table 5 showed that the water consumption and water use efficiency CK treatment during the growth period were significantly lower than those of other nitrogen application treatments in the two-year experiment, indicating that nitrogen application could significantly improve the water consumption and water use efficiency of maize. In 2022, the water consumption and water use efficiency of UNS2 treatment during the growth period were the highest, which were 453.58 mm and 32.44 kg hm^−2^ mm^−1^, respectively. CK was the lowest, at 430.84 mm and 21.77 kg hm^−2^ mm^−1^, respectively. The water consumption and water use efficiency of UNS2 treatment during the growth period were also the highest under R + S tillage mode, which were 457.89 mm and 30.88 kg hm^−2^ mm^−1^, respectively. CK was the lowest, with 428.88 mm and 22.99 kg hm^−2^ mm^−1^, respectively. The water use efficiency of R UNS2 was 5.05% higher than that of R + S UNS3.

**Table 5:** Effects of different tillage and fertilizer types on yield and water use efficiency (WUE) of maize.

Year	Treatment	Water consumption (mm)	Yield (kg hm^−2^)	water use efficiency (kg hm^−2^ mm^−1^)
2022	R-CK	430.84 ± 4.6c	9380.39 ± 120.44f.	21.77 ± 0.57e
	R-U	435.19 ± 3.05bc	11,579.21 ± 176.06e	26.61 ± 0.64d
	R-S	441.44 ± 3.7abc	12,834.44 ± 191.84d	29.07 ± 0.66c
	R-UNS1	445.75 ± 3.94ab	13,515.26 ± 143.06c	30.32 ± 0.52bc
	R-UNS2	453.58 ± 2.3a	14,712.78 ± 125.73a	32.44 ± 0.45a
	R-UNS3	446.41 ± 2.9ab	14,126.07 ± 149.22b	31.64 ± 0.37ab
	R + S-CK	428.88 ± 4.86c	9861.2 ± 106.55e	22.99 ± 0.49d
	R + S-U	433.45 ± 5.19c	11,084.3 ± 153.95d	25.57 ± 0.61c
	R + S–S	436.5 ± 4.36bc	12,329.21 ± 196.31c	28.25 ± 0.72b
	R + S-UNS1	446.64 ± 5.07abc	13,198.07 ± 155.42b	29.55 ± 0.87ab
	R + S-UNS2	457.89 ± 6.6a	14,138.3 ± 146.59a	30.88 ± 0.75a
	R + S-UNS3	453.02 ± 5.55ab	13,735.86 ± 134.01a	30.32 ± 0.65ab
Significant level(*F* value)				
Tillage(T)		ns	**	ns
Fertilization(F)		**	**	**
T × F		ns	*	ns
2023	R-CK	415.57 ± 4.79c	9201.24 ± 121.29e	22.14 ± 0.8d
	R-U	423.84 ± 5.14bc	10,892.03 ± 144.98d	25.7 ± 0.63c
	R-S	425.51 ± 4.92bc	11,708.27 ± 174.17c	27.52 ± 0.65bc
	R-UNS1	438.2 ± 3.82ab	12,637.85 ± 210.8b	28.84 ± 0.74ab
	R-UNS2	443.55 ± 3.19a	13,602.98 ± 161.93a	30.67 ± 0.62a
	R-UNS3	439.1 ± 3.16ab	12,429.2 ± 198.65b	28.31 ± 0.73ab
	R + S-CK	399.34 ± 5.87d	9101.04 ± 109.89e	22.79 ± 0.57d
	R + S-U	422.51 ± 4.93c	10,588.89 ± 158.94d	25.06 ± 0.56c
	R + S–S	427.65 ± 5.48bc	12,030.2 ± 188.75c	28.13 ± 0.63b
	R + S-UNS1	433.74 ± 4.23abc	12,849.21 ± 113.16b	29.62 ± 0.47ab
	R + S-UNS2	444.49 ± 5.16ab	13,901.88 ± 154.68a	31.28 ± 0.38a
	R + S-UNS3	446.65 ± 4.06a	13,347.81 ± 128.61b	29.88 ± 0.32ab
Significant level(*F* value)				
Tillage(T)		ns	*	ns
Fertilization(F)		**	**	**
T × F		ns	*	ns

R: rotary tillage; R + S: rotary tillage + subsoiling; CK: no fertilization; U: 300 kg hm^−2^ urea, 0 kg hm^−2^ slow-release nitrogen fertilizer; S: 0 kg hm^−2^ urea, 530.7 kg hm^−2^ slow-release nitrogen fertilizer; UNS1: 60 kg hm^−2^ urea, 424.6 kg hm^−2^ slow-release nitrogen fertilizer; UNS2: 90 kg hm^−2^ urea, 371.5 kg hm^−2^ slow-release nitrogen fertilizer; UNS3: 120 kg hm^−2^ urea, 318.4 kg hm^−2^ slow-release nitrogen fertilizer; Different alphabets indicate the significance within the same year at 5% level by LSD test. ns, not significant, (*p * > 0.05).*Significant at *p * < 0.05. **Significant at p < 0.01.

In 2023, the water consumption of UNS2 treatment was the largest, which was 443.55 mm, which was 1.01%-6.73% higher than that of the other five treatments. The water use efficiency of UNS2 treatment was the highest, which was 30.67 kg hm^−2^ mm^−1^, which was 6.34%-38.52% higher than that of the other five treatments. Under the R + S tillage method, the water consumption of UNS3 treatment was the largest, which was 446.65 mm, which was 0.48%-11.84% higher than that of the other five treatments. The UNS2 treatment had the highest water use efficiency of 31.28 kg hm^−2^ mm^−1^, which was 4.68–37.25% higher than that of the other five treatments. The water use efficiency of UNS2 was 1.98% higher than that of R UNS2.

## Discussion

In this study, the effects of the combination of slow-release nitrogen fertilizer and nitrogen fertilizer on dry matter accumulation, water use efficiency, yield, and nitrogen uptake and utilization of maize under two tillage methods were studied. The results showed that nitrogen application could improve dry matter quality, water use efficiency, yield and nitrogen use efficiency of maize. The overall performance trend under the same tillage method was UNS treatment > S treatment > U treatment > CK treatment. Compared with 2022, the dry matter accumulation, nitrogen uptake and utilization, operation, water use efficiency and yield of maize under the two tillage methods in 2023 were lower than those in 2022, and the subsoiling treatment in 2023 was better than that of rotary tillage.

Nitrogen application had a significant effect on the dry matter accumulation, yield and constituent factors of maize, and the combined application of slow-release nitrogen fertilizer and urea could ensure the nutrient supply of maize during the growth period. Studies have shown that the effect of one-time basal application of slow-release nitrogen fertilizer is similar to or significant that of fractional urea in terms of yield and its components^35^. In this experiment, all treatments were treated with one-time fertilization 15 days after emergence, and the results showed that the dry matter accumulation, yield and constituent factors of UNS treatment were significantly higher than those of S treatment and U treatment under R and R + S tillage methods, and the UNS2 treatment had the best effect, which was consistent with the results of Guo et al.^36^ and Ji et al.^37^. The accumulation of dry matter during the growth period of crops is the basis for yield formation, and the level of dry matter accumulation determines the final grain yield, which is one of the fundamental ways to increase crop grain yield^38^. In this experiment, the dry matter accumulation, spike length, grain number per spike, 100-grain quality and yield of UNS treatment were better than those of S treatment and U treatment, because the dry matter accumulation of maize showed an increasing trend of “S” curve with time, and the dry matter accumulation of ordinary urea treatment was the fastest at jointing stage, followed by large flare stage. However, after the tasseling stage, it was significantly lower than that of the slow-release nitrogen fertilizer combined treatment, and although the common urea had a fast fertilizer effect in the early growth stage, it could provide good fertility, promote the nutrient absorption of maize roots, and improve the dry matter accumulation of maize plants (Fig. 2 R–U treatment in the JT period), but due to nitrogen loss, it could not effectively provide fertility in the late growth stage, which led to the weakening of the nutrient absorption capacity of maize roots, and the dry matter accumulation and yield were lower than those of the combined application of slow-release nitrogen fertilizer and urea. However, due to the time required to release fertility by slow-release nitrogen fertilizer alone, the supply of early growth fertility was insufficient, resulting in defertilization (Fig. 3 R–S treatment in JT period). The combination of slow-release nitrogen fertilizer and urea can well solve the shortcomings of slow nitrogen release and insufficient nutrients in the early stage of crop growth, and ensure the fertility supply of crops in the whole growth season, which is similar to the conclusion of Wang et al.^39^ that slow-release nitrogen fertilizer can improve the dry matter accumulation of summer maize, which is mainly reflected in the conclusion after the silking period.

Studies have shown that nitrogen fertilization has a significant effect on water uptake and utilization of maize, and can improve water use efficiency^40^. In this experiment, the water use efficiency of UNS treatment > S treatment > U treatment > CK treatment under the two tillage methods, which is consistent with the conclusion that controlled-release urea can improve the water use efficiency of crops by Zheng et al.^41^ and Hu et al.^42^, and the water use efficiency of UNS2 treatment reached the highest level. The reason is that the application of slow-release nitrogen fertilizer can ensure that maize has sufficient nutrients in the later growth stage, and can still maintain high root volume and metabolic enzyme activity in the later stage, which can improve the ability of maize to absorb water, thereby significantly improving its water use efficiency, while urea single application treatment can lead to denitrification in the late growth stage of maize under the one-time fertilization mode, thereby limiting its growth and development and reducing water use efficiency, which is consistent with the results of Lv et al.^43^

For a long time, with the increase of crop yields, the use of nitrogen fertilizers has increased year by year, which has exacerbated nitrogen loss and environmental pollution, and caused an increase in costs^44^. Studies have shown that the combination of slow-release nitrogen fertilizer and urea can alleviate this situation, reduce nitrogen loss, improve nitrogen accumulation, utilization, and operation, and save costs ^45^. In this experiment, the nitrogen accumulation and nitrogen uptake and utilization of aboveground parts of maize plants were treated by UNS treatment > S treatment > U treatment, among which UNS2 treatment was the best, which was consistent with the results of Zhou et al.^46^ and Wang et al.^47^. Compared with the S treatment and U treatment, UNS treatment can solve the problem of insufficient nutrient supply in the early or late stage of maize growing season, promote nitrogen accumulation in shoots, improve nitrogen use efficiency of maize, and improve nitrogen harvest index, which is consistent with the results of Guo et al.^48^. At the same time, in this study, the nitrogen uptake of vegetative organs stored in the vegetative organs of maize plants at the tasseling stage was greater than that at the maturity stage, indicating that the nitrogen stored in the vegetative organs was transferred to the grain after the tasseling stage, and the nitrogen in the UNS treatment had the highest amount of nitrogen to the grain and the highest efficiency to the grain, and the UNS2 treatment was the best. This is due to the fact that the nitrogen in the grain comes from two parts, one part is the nitrogen absorbed and stored in the vegetative organs before the tasseling stage and transferred to the grain after the tasseling stage, and the other part is the nitrogen absorbed and assimilated by the plant after the tasseling stage. Therefore, the combination of urea and slow-release nitrogen fertilizer can improve nitrogen accumulation and nitrogen use efficiency, so that more nitrogen can be transferred to the grain, thereby increasing yield, which is similar to the results of Wang et al.^49^

Compared with rotary tillage, subsoiling is a commonly used conservation tillage measure, which has less disturbance to the soil in the tillage layer than traditional rotary tillage, can increase the depth of the tillage layer, break the bottom layer of the plough, reduce the soil bulk density, promote root growth, improve water use efficiency, increase the dry matter accumulation of crops, and promote the accumulation, absorption, utilization and transport of nitrogen in the aboveground parts, thereby increasing the yield^50,51^. The results of Yin et al.^52^ and Liu et al.^53^ showed that the combined application of slow-release nitrogen fertilizer and urea could reduce the maximum depth of soil nitrate nitrogen, increase the nitrate nitrogen content in the 0–40 cm soil layer, and decrease the nitrate nitrogen content in the 40–100 cm soil layer, reducing the nitrogen loss, which was conducive to the absorption, utilization and operation of nitrogen, and improved the nitrogen utilization efficiency, and concluded that the comprehensive effect was the best when the ratio was 7:3 (slow-release nitrogen fertilizer: urea). In this experiment, the two tillage methods of subsoiling and rotary tillage were interacted with slow-release nitrogen fertilizer and urea, and the subsoiling was 50 cm, which not only made the spatial distribution of roots reasonable, but also met the spatial requirements of nitrogen absorption in maize. Due to the slow release of nitrogen, slow-release nitrogen fertilizer significantly improves soil nitrogen supply in the late growth stage and meets the time requirement of crop nitrogen demand. The results showed that the dry matter accumulation, water use efficiency, yield, nitrogen uptake and utilization and operation of the subsoiling treatment in 2022 were slightly lower than those of the rotary tillage treatment, which was slightly different from the results of Cao et al.^54^ and Ji et al.^55^, because the plots were not all disturbed in the first year of subsoiling, and the bottom layer of the soil plough was not completely broken, so the effect of subsoiling treatment in 2022 was slightly lower than that of rotary tillage treatment. In 2023, after two consecutive years of subsoiling, 80% of the soil in the plot was disturbed, the breaking effect of the plough bottom layer was significantly better than that in 2022, the soil compactness and soil bulk density were reduced, the spatial distribution of roots was more reasonable, the biomass of roots in the soil was improved, the absorption of nutrients and water was promoted, the soil water use efficiency was improved, the ear length, the number of grains per spike and the weight of 100 grains were increased, and the nitrogen absorption, utilization and operation were promoted, thereby promoting the increase of corn yield, and the subsoiling treatment was better than the rotary tillage treatment. Therefore, on the basis of subsoiling, the combination of slow-release nitrogen fertilizer and urea can better meet the space and time requirements of root absorption, and can improve the dry matter accumulation, water use efficiency, nitrogen accumulation, absorption, utilization and transport of spring maize, thereby increasing yield, which is consistent with the research results of Zhou et al.^56^ and Hu et al.^57^. However, the rainfall during the growth period in 2023 was significantly lower than that in 2022, which limited the growth and development of maize roots, and reduced the ability of roots to absorb nutrients and water in the soil, which led to dry matter accumulation, water use efficiency, nitrogen uptake, utilization and transport, yield and its components under the two tillage methods of R and R + S in 2023 were slightly lower than those in 2022.

## Conclusion

This study showed that after two years of subsoiling to break the bottom layer of the plough, the subsoiling effect was significant, and the subsoiling treatment could effectively improve the yield, nitrogen uptake and utilization, water use and dry matter accumulation of maize compared with rotary tillage treatment. UNS can effectively ensure the nutrient supply of maize during the growth period, so as to achieve the effect of increasing yield. Among them, UNS2 has the best treatment effect and the highest yield, which is the most suitable fertilizer ratio, and should be demonstrated and promoted in a large area. After many years of long-term tracking and positioning experiments, the effect of breaking the bottom layer of the plough will be better, and the next research will continue to focus on multi-year subsoiling and rotary tillage, in order to provide a theoretical and scientific basis for selecting suitable tillage mode and optimal fertilization ratio in areas with similar ecological types.

## Data Availability

The original contributions presented in the study are included in the article, further inquiries can be directed to the corresponding author.
